# Alleviation of Ammonium Toxicity in *Salvia splendens* ‘Vista Red’ with Silicon Supplementation

**DOI:** 10.3390/toxics10080446

**Published:** 2022-08-03

**Authors:** Jinnan Song, Jingli Yang, Byoung Ryong Jeong

**Affiliations:** 1 Department of Horticulture, Division of Applied Life Science (BK21 Four Program), Graduate School of Gyeongsang National University, Jinju 52828, Korea; jinnansong93@gmail.com (J.S.); yangmiaomiaode@gmail.com (J.Y.); 2 Institute of Agriculture and Life Science, Gyeongsang National University, Jinju 52828, Korea; 3 Research Institute of Life Science, Gyeongsang National University, Jinju 52828, Korea

**Keywords:** acidic stress, antioxidant enzymes, bedding plants, photosynthesis, cation uptake, lipid peroxidation, nitrogen (N) nutrient, reactive oxygen species (ROS)

## Abstract

Ammonium (NH_4_^+^) toxicity seriously hampers the yield and quality of salvia plants because most varieties or sub-species are highly sensitive to NH_4_^+^. Silicon (Si) is an alternative that is used to minimize these disturbances and maintain better growth under NH_4_^+^ toxicity. Nevertheless, the mitigatory effects of Si on NH_4_^+^-stressed salvia are unknown. Therefore, this study was carried out to determine how Si assists to alleviate the NH_4_^+^ toxicity degree in salvia. To this end, salvia plants were cultivated in a controlled environment supplied with a constant N (nitrogen) level (13 meq·L^−1^) in the form of three NH_4_^+^:NO_3_^−^ ratios (0:100, 50:50, 100:0), each with (1.0 meq·L^−1^) or without Si. Physiological disorders and typical NH_4_^+^ toxicity symptoms, as well as interrupted photosynthesis, were observed in the 100% NH_4_^+^-treated plants. Furthermore, cation uptake inhibition and oxidative damage were also imposed by the 100% NH_4_^+^ supply. In contrast, in the presence of Si, the NH_4_^+^ toxicity degree was attenuated and plant growth was ensured. Accordingly, the NH_4_^+^ toxicity appearance ratio decreased significantly. Furthermore, Si-treated plants showed an ameliorated photosynthetic ability, elevated internal K and Ca levels, and enhanced antioxidative capacity, as reflected by improved major antioxidant enzyme activities, as well as diminished accumulation of ROS (reactive oxygen species) and MDA (malondialdehyde). Our findings enlightened the agronomic importance of additional Si to nutrient solutions, especially pertaining to bedding plants at risk of NH_4_^+^ toxicity.

## 1. Introduction

Nitrogen (N) is a fundamental nutrient that determines plant biomass production and crop gains. The absorption and assimilation of N by plants are predominately in the forms of ammonium (NH_4_^+^) and nitrate (NO_3_^−^) [1]. The acquisition processes of these two forms regarding the energetic and biochemical aspects are shown to be distinct [2]. NH_4_^+^ assimilation is more metabolically efficient than NO_3_^−^ assimilation because only NH_4_^+^ can be incorporated into organic compounds, which should theoretically mean that NH_4_^+^ is the preferred N form [3,4].

Paradoxically, it appears that most plants manifest poor growth performance when a high NH_4_^+^ level is unintentionally presented or as the exclusive N source [5,6]. Indeed, an excessive supply of NH_4_^+^ (usually millimolar level) for higher plants leads to the suppression of plant growth and development. Abundant ammonium assimilation causes acidic stress, which is considered the primary cause of NH_4_^+^ toxicity [7]. This phenomenon can be developed and identified in many plant species, ranging from crops to vegetables [3,8,9]. Consequently, various characteristics concerning physiology and biochemistry are severely altered: inhibition of the regulation of intracellular pH, disruption of photosynthetic ability, impaired cation influxes (in particular K^+^, Ca^2+^, Mg^2+^) and ion equilibrium, and interference with the overproduction of reactive oxygen species [10]. These summed factors during NH_4_^+^ toxicity contribute to detrimental visual symptoms that are characterized by reduced plant growth, leaf chlorosis, and necrosis, as well as stunted roots [10,11].

Salvia (*Salvia splendens* F. Sellow ex Roemer & J.A. Schultes) is a widely cultivated herbaceous bedding plant in the *Salvia* genus. It is extensively utilized for culinary purposes and essential oil extraction [12]. Ornamentally, it is valued for the tender flowers and aromatic foliage [13]. However, according to pioneer reports, most of the salvia varieties or sub-species are determined to be extremely sensitive to NH_4_^+^ [6,14,15]. Therefore, the breeding of salvia was highly hindered by the toxic levels of NH_4_^+^ input. Thereafter, more optimal fertilization strategies regarding agronomic and agricultural attempts were warranted. Meanwhile, silicon supplementation benefits salvia growth in harsh environments [16]. Moreover, the supplementations of silicon nutrition can reverse or reduce the NH_4_^+^ toxicity stress degree [17].

As the second-most abundant element after oxygen in the Earth’s crust, silicon (Si) was recognized to be a ‘quasi-essential’ inorganic constituent for higher plants [18]. Outstandingly, the beneficial impacts of Si application were witnessed in plants under biotic [19,20] and abiotic stresses [21,22]. The improvement in photosynthetic capacity due to Si is correlated with the formation of a subcuticular double layer on the leaf epidermis, resulting in greater light interception and absorption abilities and lower water loss via transpiration [17,23]. Furthermore, Si is believed to overcome the depletion of ions caused by the NH_4_^+^ toxicity because Si accumulated in roots promotes the hydraulic movement for the selective influx of cations [24]. On the one hand, Si is associated with the rigidity and integrity of cell walls, which contributes to the maintenance of chloroplast structures and diminishment of malondialdehyde (MDA), which is defined as the end product of lipid peroxidation [17,25]. On the other hand, Si assists in the modulation of activities of key antioxidant enzymes under oxidative stresses, accordingly detoxifying the over-accumulation of reactive oxygen species (ROS, i.e., O_2_^·^^−^, H_2_O_2_) [21,26,27]. Regarding the ameliorative role of Si against NH_4_^+^ toxicity, relevant work in salvia is highly insufficient.

Therefore, to determine the alleviatory effects of Si supplementation on NH_4_^+^-stressed salvia plants, the growth attributes, photosynthetic ability, key ion accumulations, antioxidative capacity (major antioxidant enzymes activities), ROS content, and lipid peroxidation level in response to an increasing NH_4_^+^ nutrition supply were evaluated in the current study. To the best of the authors’ knowledge, this is the first attempt to unveil the mitigator role of Si against NH_4_^+^ toxicity in salvia.

## 2. Materials and Methods

### 2.1. Plant Materials, Treatments, and Experimental Conditions

Seeds of salvia (*Salvia splendens* ‘Vista Red’, provided by PanAmerica Seed company, West Chicago, IL, USA) were sown in 200-cell plug trays containing BVB medium (Bas Van Buuren Substrate, EN-12580, De Lier, The Netherlands) moistened with tap water. The seeds were germinated in a mist propagation bench under a naturally lighted greenhouse condition at Gyeongsang National University (35°86′ N, 128°03′ E, Jinju, Korea) from 20 October 2021 to 1 November 2021. Seedlings were cultivated with MNS (multipurpose nutrient solution) [9] for another week (18 days after sowing (DAS)) until two true leaves fully expanded. The composition of the MNS was as follows (macronutrients in me·L^−1^): 6.0 Ca(NO_3_)_2_·4H_2_O, 2.0 MgSO_4_·7H_2_O, 5.0 KNO_3_, and 2.0 NH_4_H_2_PO_4_; and (micronutrients in μmol·L^−1^): 20 H_3_BO_3_, 0.5 CuSO_4_·5H_2_O, 10 Fe-EDTA, 10 MnSO_4_·4H_2_O, 0.5 H_3_MoO_4_, and 4.0 ZnSO_4_·7H_2_O. Furthermore, the seedlings were grown in a hydroponic substrate system by using a commercial BVB medium (Bas Van Buuren Substrate, EN-12580, De Lier, The Netherlands) consisting of peat, coconut, and perlite.

The seedlings with similar morphologies were screened out to new 200-cell plug trays (plug tray information can be found on https://www.amazon.com/Seedling-Starter-Trays-Extra-Strength/dp/B01GBBZU2Y accessed on 26 November 2021), which were subjected to running tap water for three days (21 DAS) to leach all the nutrition and maintained in a controlled alternating diurnal regime with 10 h light (10.8 daily light integral (DLI), white LED) and 14 h darkness at an air-conditioned temperature of 23 °C/18 °C and 60 ± 10% relative humidity. Subsequently, three NH_4_^+^:NO_3_^−^ ratios (0:100, 50:50, and 100:0) at a constant N supply (13 me·L^−1^) were formulated based on the MNS, which corresponded with an optimized Si concentration (1.0 me·L^−1^) sourced from K_2_SiO_3_ according to our lab’s previous publication [28] or without Si (Table 1). Excessive potassium sourced from K_2_SiO_3_ in 0:100, 50:50, and 100:0 solutions were reduced using KNO_3_, K_2_SO_4_, and K_2_SO_4_, respectively, and the resultant losses of nitrate and sulfate were balanced with the addition of nitric and sulfuric acid. Therefore, a total of six solutions consisting of 0:100 NH_4_^+^:NO_3_^−^ Si (−), 50:50 NH_4_^+^:NO_3_^−^ Si (−), 100:0 NH_4_^+^:NO_3_^−^ Si (−), 0:100 NH_4_^+^:NO_3_^−^ Si (+), 50:50 NH_4_^+^:NO_3_^−^ Si (+), and 100:0 NH_4_^+^:NO_3_^−^ Si (+) were adopted for the treatments. For each treatment, three repetitions involving a total of 60 plants were laid out with a 2 × 3 factorial scheme in a completely randomized design. Moreover, blank columns of the plug trays between different treatments were intentionally kept.

### 2.2. Measurement of Plant Growth Parameters and Destructive Sampling

The treatment solutions were applied every two days for 15 days (36 DAS). Subsequently, the plants were demounted out of the plug tray and the medium was washed off (39 DAS). Whole-plant fresh weight (surface-blotted with absorbent paper) and dry weight (kept in an air-forced oven at 60 °C for 72 h) were measured with an electronic balance. Lengths of shoots and tap roots, as well as leaf lengths and widths, were measured with a metal ruler. The leaf area was determined with a leaf area analysis meter (Li-3000, Li Cor Inc., Lincoln, NE, USA). The two topmost true leaves from each plant were individually sampled, immediately frozen in liquid nitrogen, and preserved at −70 °C for further analysis.

### 2.3. Calculation of the Ammonium Toxicity Ratio (%)

The toxicity ratio per replicate was determined using the following equation:

(1) $$\mathrm{Ammonium}\;\mathrm{toxicity}\;\mathrm{ratio}\;\left(\%\right)=\frac{\mathrm{No}.\;\mathrm{of}\;\mathrm{plants}\;\mathrm{with}\;\mathrm{ammonium}\;\mathrm{toxicity}\;\mathrm{symptoms}}{20}\times 100\%$$

where ‘20’ is the number of plants per replicate.

### 2.4. Estimation of the Photosynthetic Capacity

The photosynthetic ability was estimated herein using the chlorophyll content and maximum photochemical quantum yield of PSII (Fv/Fm value). Specifically, the total chlorophyll concentration (sum of chlorophyll a and b) was determined following a protocol proposed by Sims and Gamon [29]. Fv/Fm was detected using a FluorPen FP 100 (Instruments of Photon Systems, Drásov, Czech Republic).

### 2.5. Determinations of Si, K, Ca, and Mg Concentrations

Ion contents of Si, K, Ca, and Mg in the plants were determined with an inductively coupled plasma (ICP) spectrometer (Perkin Elmer, Rodgau, Germany).

Samples for the ICP analysis were prepared according to a slightly modified procedure [30]: Fresh whole plants were placed in an air-forced oven (Jeio Technology Co. Ltd., Daejeon, Korea) at 70 °C until they reached a constant weight. Afterward, 100 mg of dried samples were ashed using a Nabertherm muffle furnace programmed at 525 °C for 120 min. The ash specimen was digested with 5 mL 25% HCl and adjusted to a final volume of 30 mL by adding 25 mL of distilled water. These incubations were performed at ambient temperature.

### 2.6. Analysis of Antioxidant Enzyme Activities in Leaf Samples

Frozen leaf samples were finely ground in a pre-cooled mortar over an ice bath. Then, 100 mg of the fine powder was quickly weighed and homogenized in an extraction medium (50 mM of PBS, 1 mM of EDTA, 2% polyvinylpyrolidone, 0.05% triton-X at pH = 7.0). The mixture was subjected to centrifugation (13,000 rpm, 20 min, 4 °C) for the acquirement of the supernatant, which was used afterward for the total soluble protein quantification and enzymatic measurements.

The total protein content was quantified using Bradford’s reagent [31]. Analysis of the enzyme activities was carried out over an ice bath following the method described by Biju [32]. Superoxide dismutase (SOD) activity was assayed based on NBT (nitroblue tetrazolium) reduction [33]. A method based on ascorbate oxidation was adopted for measuring the ascorbate peroxidase (APX) activity [34]. A rapid and sensitive procedure that utilized H_2_O_2_ decomposition was employed for determining the catalase (CAT) activity [35]. Guaiacol peroxidase (GPX) activity was estimated based on the reaction of guaiacol oxidation [36]. Dehydroascorbate reductase (DHAR) activity was measured according to a methodology proposed by Nakano [34]. Glutathione reductase (GR) activity was determined as presented by Mavis [37].

### 2.7. Quantifications of O_2_^·^^−^, H_2_O_2_, MDA, and Carotenoids in Leaf Samples

The O_2_^·^^−^ (superoxide) level was quantified following an approach proposed by Wu using hydroxylamine oxidization [38]. The H_2_O_2_ (hydrogen peroxide) concentration was colorimetrically determined using a protocol according to Mukherjee [39]. The level of lipid peroxidation was monitored in terms of the MDA (malondialdehyde) content, which was measured based on the TBA (thiobarbituric acid) reaction [40]. The detailed procedure can be seen in Li’s publication [41]. The carotenoid contents were assessed using the identical sample preparation methods as for chlorophyll [29] but the absorbance was spectrophotometrically read at 440 nm.

### 2.8. Statistical Analysis and Graphing

All the data presented are the means ± standard deviations (SDs) of at least three biological replicates. They were subjected to the Duncan’s multiple range test or the two-sided Student’s *t*-test to determine the significant differences at *p* = 0.05 using the SAS statistical software (V. 8.2, Cary, NC, USA) and GraphPad Prism 8.0 (GRAPH PAD software Inc., San Diego, CA, USA), respectively. And the *F*-test was performed to find the significance between treatments using SAS statistical software 8.2. The PCA graph was plotted using Origin 2022 software (Origin Lab Corp., Northampton, MA, USA) for the variability identification of antioxidative capacity results.

## 3. Results

### 3.1. Effects of the Three NH_4_^+^:NO_3_^−^ Ratios and Si Supplementation on the Plant Growth Attributes

**Figure 1:**
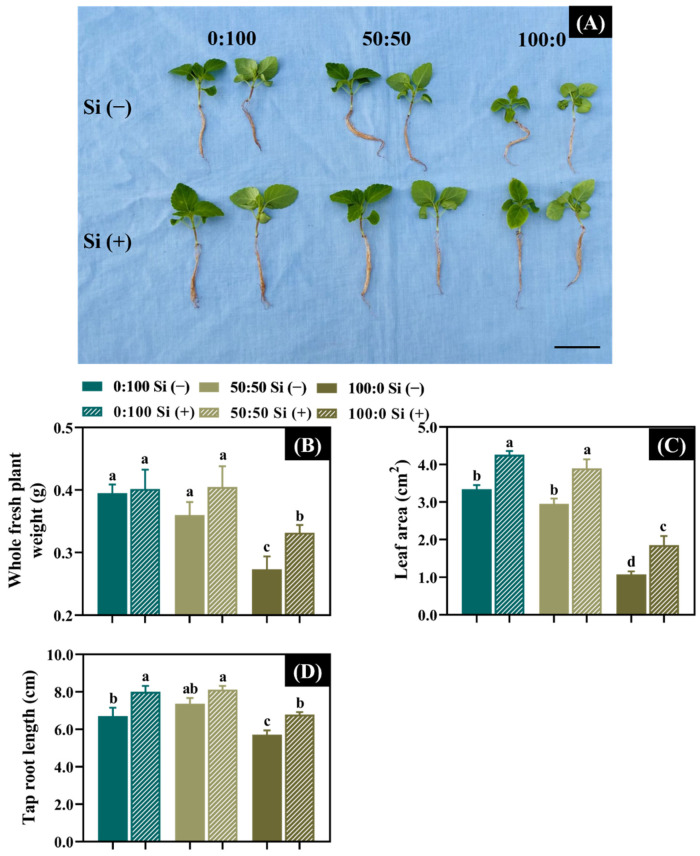
Effects of the three NH_4_^+^:NO_3_^−^ ratios with or without Si on (**A**) salvia growth attributes in terms of the (**B**) whole fresh plant weight, (**C**) leaf area, and (**D**) tap root length. A plant pair with similar growth was employed to present the identical treatment in (**A**). Values are given as the average ± standard deviation (SD) of *n* = 6 replicates. Different lowercase letters (generated by Duncan’s multiple range test) were used for the statistical significances. Scale bar: 2 cm.

In the absence of Si nutrition, the plant growth characteristics were shaped in contrasting manners by the different N forms. Similar growth was obtained between the plants cultivated with 0:100 and 50:50 NH_4_^+^:NO_3_^−^ nutrition, whereas plant growth was remarkably slowed in solely NH_4_^+^-treated plants (Figure 1A). Indeed, this observation was evidenced in terms of the whole plant weight, leaf area, and tap root length: the whole plant weight, leaf area, and tap root length were significantly reduced by 25, 63.4, and 22.4%, respectively, when the external NH_4_^+^ supply increased from 50 to 100% (Figure 1B–D).

In contrast, as expected, the plant growth was dramatically improved or ameliorated after Si was individually supplemented (Figure 1A). Specifically, in comparison with Si (−), plants grown in 0:100, 50:50, and 100:0 NH_4_^+^:NO_3_^−^ regimes nourished with Si respectively gained 1.8, 12.5, and 20.9% greater whole fresh plant weights (Figure 1B). The individual addition of Si to 0:100, 50:50, and 100:0 NH_4_^+^:NO_3_^−^ solutions significantly enhanced the leaf area by 27.5, 32.2, and 72.9%, respectively (Figure 1C). The tap root length of plants cultured with 0:100 and 100:0 NH_4_^+^:NO_3_^−^ solutions were also markedly improved by 19.4 and 18.5%, respectively, after Si nutrition was added (Figure 1D).

Concomitantly, we obtained strong interactions between the provided N forms and Si application regarding dry weight, shoot length, and leaf length and width, as depicted by the *F*-test results (Table 2).

### 3.2. NH_4_^+^ Toxicity Ratio as Influenced by Si Application

**Figure 2:**
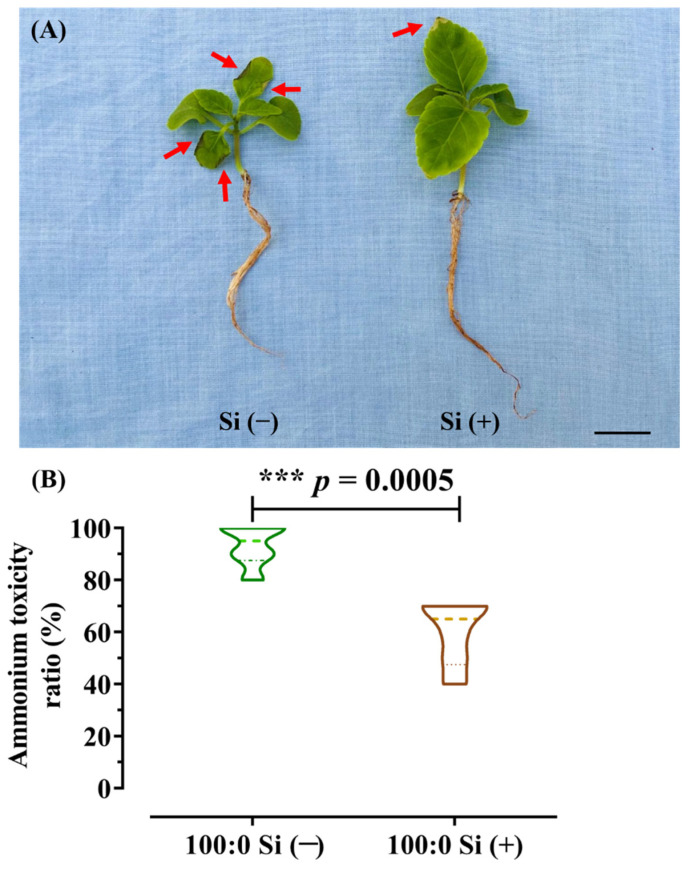
(**A**) Plant growth status and (**B**) NH_4_^+^ toxicity appearance ratio (%) in response to 100:0 NH_4_^+^:NO_3_^−^ nutrient solution supplemented with or without Si. Data displayed is the mean ± standard deviation (SD) of *n* = 6 replicates. Statistical significances (*** *p* < 0.001) between Si (−) and Si (+) were determined using a two-tailed Student’s *t*-test. Red arrows point to the typical NH_4_^+^ toxicity symptoms in foliage. Scale bar refers to 2 cm.

Notably, the salvia plants subjected to 100% NH_4_^+^ nutrition developed NH_4_^+^ toxicity symptoms, irrespective of the Si application, as physiologically characterized by visual chlorosis and foliage necrosis accompanied by burned tips (Figure 2A).

However, Si supplementation to the 100:0 NH_4_^+^:NO_3_^−^ solution drastically ameliorated the plant growth status. Consequently, statistically, the NH_4_^+^ toxicity appearance ratio after the Si addition significantly decreased (*p* = 0.0005) from 93.3 to 60% (Figure 2B).

### 3.3. Photosynthetic Ability as Affected by the N Form and Si Supplementation

**Figure 3:**
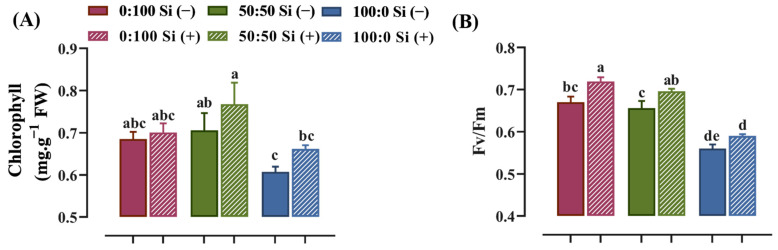
Photosynthetic capacity as characterized by (**A**) chlorophyll content and (**B**) Fv/Fm value in response to N forms with or without Si supplementation. Data are given as the mean ± standard deviation (SD) of *n* ≥ 4 replicates. Different lowercase letters over bars indicate statistical significance according to Duncan’s multiple range test at *p* = 0.05.

The photosynthetic capacity was visibly affected by the N form, which influenced the chlorophyll content and quantum efficiency of the photosystem II photochemistry, where the latter can be assessed using the Fv/Fm value. The salvia plants cultured with a mixture of NH_4_^+^ and NO_3_^−^ possessed a higher chlorophyll content. More chlorophyll degradation was observed when the external NH_4_^+^ supply increased from 50 to 100% (Figure 3A). In a similar manner to the Si-deficient plants, a progressively reduced Fv/Fm value was observed in response to an enhanced external NH_4_^+^ supply. Specifically, salvia plants treated with 0:100 NH_4_^+^:NO_3_^−^ solution showed 2.1 and 19.6% higher Fv/Fm values, respectively, compared with those cultured in 50:50 and 100:0 NH_4_^+^:NO_3_^−^ regime (Figure 3B).

More importantly, the plants upgraded their photosynthetic abilities to varying extents in response to Si supplementation, especially for the solely NH_4_^+^-treated plants. The addition of Si to the plants grown in the 100:0 NH_4_^+^:NO_3_^−^ solution markedly enhanced the total chlorophyll content by 10% (Figure 3A). In parallel, in comparison with the 100:0 NH_4_^+^:NO_3_^−^ Si (−), the Fv/Fm value was significantly higher for the plants nourished with Si (Figure 3B).

### 3.4. The Accumulation of Silicon (Si), Potassium (K), Calcium (Ca), and Magnesium (Mg)

The Si supplementations for salvia plants cultivated under different treatments not only distinctly influenced the internal Si level, but also the uptake of certain NH_4_^+^-associated cations, such as K^+^, Ca^2+^, and Mg^2+^.

**Figure 4:**
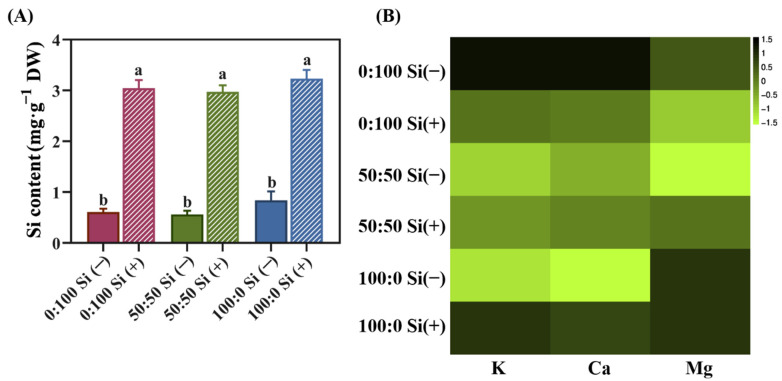
Si, K, Ca, and Mg contents in plants as affected by the N form with or without Si supplementation. (**A**) Si content in salvia plants subjected to different treatments; data are given as the mean ± standard deviation (SD) of *n* = 3 technical replicates. Significant differences are shown using different lowercase letters, which were determined using Duncan’s multiple range test at *p* = 0.05. (**B**) Concentrations of K, Ca, and Mg in plants were compared and illustrated via a heatmap; the original data was averaged, log2-normalized, and clustered following the criterion by Chen [42].

Obviously, the external Si application significantly increased the accumulation of Si in plants, regardless of the NH_4_^+^:NO_3_^−^ ratios. Specifically, the incorporation of Si in the 50:50 NH_4_^+^:NO_3_^−^ solution provided a notably improved 4.3-fold higher amount of Si to the plants; likewise, the Si content in solely NO_3_^−^-treated and NH_4_^+^-treated salvia plants respectively enhanced the internal Si content by 3.4- and 3.3-fold after Si was applied (Figure 4A). Notably, no statistical difference was found between the different N forms, regardless of the Si supply (Figure 4A).

Thereafter, for the Si-deficient plants, we detected a gradual reduction in internal K and Ca levels in response to an increasing external level of NH_4_^+^ supply from 0 to 100%. However, as expected, except for the monitored Mg content in exclusively NH_4_^+^-cultured plants, Si supplementation dramatically enhanced the K and Ca concentrations in salvia plants grown in both the 50:50 and 100:0 NH_4_^+^:NO_3_^−^ regimes (Figure 4B). Surprisingly, the K, Ca, and Mg contents did not increase for the solely NO_3_^−^-treated plants in the presence of Si (Figure 4B).

### 3.5. Responses of Antioxidant Capacity to N Forms and Si Application

When the plants suffered from external stress, the oxidative protective system regarding antioxidant enzymes would be triggered to lower the stress degree. During this process, the activity of antioxidant enzymes was upregulated to produce enhanced antioxidant capacity. The activities of major antioxidant enzymes concerning SOD (superoxide dismutase), CAT (catalase), APX (ascorbate peroxidase), GPX (guaiacol peroxidase), GR (glutathione reductase), and DHAR (dehydroascorbate reductase) were therefore quantified.

**Figure 5:**
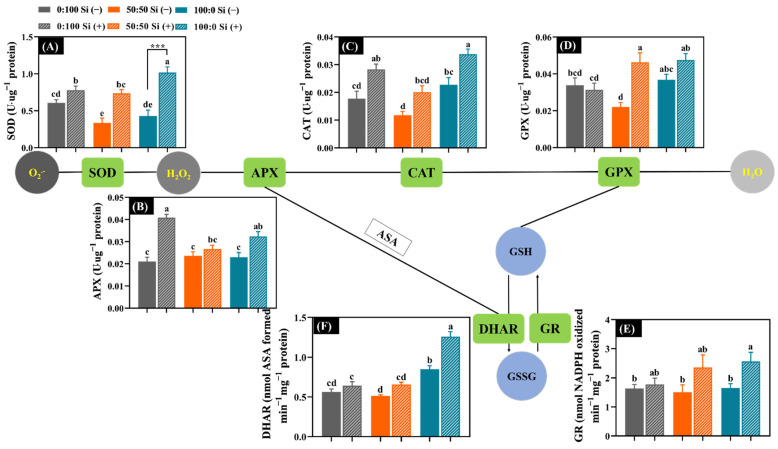
The effects of the N form with or without Si supplementation on the antioxidant capacity implicated the activity of key antioxidant enzymes. The activities of (**A**) superoxide dismutase (SOD), (**B**) ascorbate peroxidase (APX), (**C**) catalase (CAT), (**D**) guaiacol peroxidase (GPX), (**E**) glutathione reductase (GR), and (**F**) dehydroascorbate reductase (DHAR). The GSH, glutathione; GSSG, glutathione disulfide; and ASA, ascorbate. *n* ≥ 4 technical replicates were averaged and statistically analyzed following the Duncan’s multiple range test at *p* = 0.05 and denoted by different lowercase letters. Statistical significances (*** *p* < 0.001) of SOD activity in solely NH_4_^+^-treated plants between Si (−) and Si (+) were determined using the two-tailed Student’s *t*-test. Error bars denote mean ± standard deviation (SD).

When the external NH_4_^+^ level increased from 50 to 100%, Si-deficient plants gradually improved the activity of antioxidant enzymes; similarly, the addition of 50% NH_4_^+^ to a solely NO_3_^−^ solution to varying extents decreased the enzyme activities, except for APX (Figure 5B). By contrast, the added Si significantly boosted the enzyme activities, independent of the NH_4_^+^:NO_3_^−^ ratios (Figure 5). Outstandingly, the addition of Si to a 100:0 NH_4_^+^:NO_3_^−^ solution produced a 1.4-fold higher SOD activity relative to 100:0 NH_4_^+^:NO_3_^−^ Si (−) (***, *p* = 0.0003) (Figure 5A). Concomitantly, and also significantly, less pronounced increases were observed for other enzyme activities in the presence of Si: supplementations of Si to the plants reinforced the activities of APX, CAT, GPX, GR, and DHAR, regardless of the NH_4_^+^:NO_3_^−^ ratios (Figure 5B–F).

### 3.6. Oxidative Damage as Affected by the Three NH_4_^+^:NO_3_^−^ Ratios and Si Supplementations

To better understand the responses of antioxidant capacity to the N form and Si application, the oxidative damage in terms of the ROS accumulation (O_2_^·^^−^ and H_2_O_2_) and lipid peroxidation (MDA, chlorophyll, and carotenoids) were evaluated.

**Figure 6:**
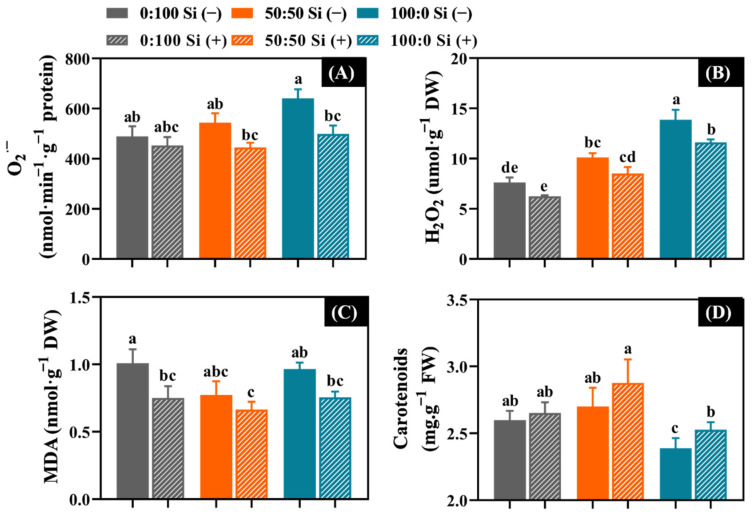
The impact of the N form with or without Si supplementation on the content of (**A**) superoxide anion (O_2_^·^^−^), (**B**) hydrogen peroxide (H_2_O_2_), (**C**) malondialdehyde (MDA), and (**D**) carotenoids. *n* = 6 technical replicates were averaged and statistically analyzed using the Duncan’s multiple range test at *p* = 0.05. Significant differences were denoted by different lowercase letters. Error bars indicate mean ± standard deviation (SD).

NH_4_^+^ stress rapidly increased the O_2_^·^^−^ (17.9%), H_2_O_2_ (37.4%), and MDA (26.0%) contents, while reducing the carotenoids (11.9%) in contrast to the plants cultivated with 50:50 NH_4_^+^:NO_3_^−^ (Figure 6). In addition, a gradual enhancement was found regarding the O_2_^·^^−^ and H_2_O_2_ accumulation as the external NH_4_^+^ increased from 0 to 100% (Figure 6A,B,D).

As expected, the supplementation of Si to varying extents decreased the ROS accumulation and lipid peroxidation, regardless of the NH_4_^+^:NO_3_^−^ solutions considered (Figure 6). Outstandingly, after Si supplementation, the solely NH_4_^+^-treated salvia remarkably diminished the accumulation of O_2_^·^^−^ (22.2%), H_2_O_2_ (16.4%), and the production of MDA (22.7%); meanwhile, a significant increase was observed in the carotenoids content (6.3%).

### 3.7. Responses between Antioxidant Capacity and Si Supplementation Are Supported by PCA Analysis

To visualize the influences of different NH_4_^+^:NO_3_^−^ ratios with and without Si on the antioxidant enzymes and oxidative damage, as well as the relationship between antioxidant capacity and Si supplementations, PCA (principal component analysis) based on the antioxidant enzyme activities, ROS accumulation (O_2_^·^^−^ and H_2_O_2_), and lipid peroxidation data set was performed.

**Figure 7:**
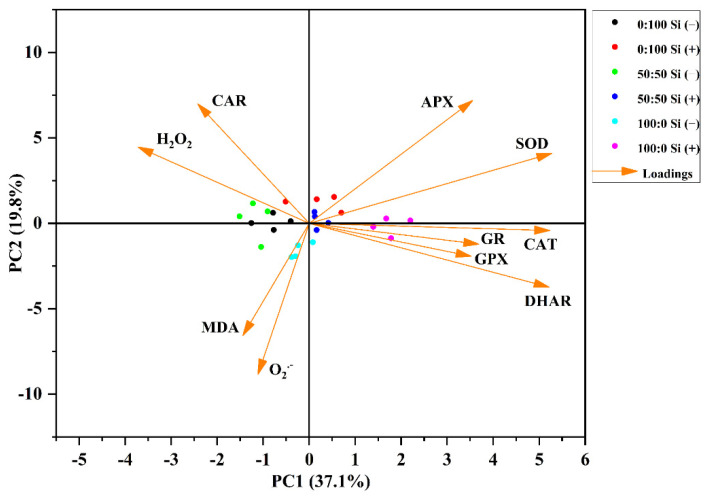
Multivariate data analysis using principal component analysis (PCA) of indices involved in antioxidant defense and oxidative damage. SOD: superoxide dismutase, APX: ascorbate peroxidase, CAT: catalase, GPX: guaiacol peroxidase, GR: glutathione reductase, DHAR: dehydroascorbate reductase, O_2_^·^^−^: superoxide anion, H_2_O_2_: hydrogen peroxide, MDA: malondialdehyde, CAR: carotenoids.

The computed model produced the related parameters along the first two principal components that captured 56.9% (PC1: 37.1%, PC2: 19.8%) of the total observed data variability (Figure 7). The majority of the Si-deficient plants ‘Si (−)’ were distributed in the left quadrants of the PC1 scatter plot, whereas Si-sufficient plants ‘Si (+)’ were separated to the right quadrants of the PC1 scatter plot, which indicated that Si (−) plants were negatively correlated with the Si (+) plants on these mentioned traits (Figure 7 ‘PC1’).

In addition, solely NH_4_^+^-treated plants with Si supplementations displayed a higher activity of major antioxidant enzymes (CAT, GR, GPX, DHAR, SOD, APX), which had strong negative correlations with H_2_O_2_ (Figure 7 ‘PC1’). In a similar context, APX and SOD were negatively correlated with MDA and O_2_^·^^−^ (Figure 7 ‘PC2’).

## 4. Discussion

The NH_4_^+^-spiked supply inevitably instigated the over-assimilation of NH_4_^+^ by plants, resulting in plant tissue acidification, which is the primary cause of NH_4_^+^ toxicity [7]. It not only curtailed the shoot and root growth and the biomass production but also produced certain visual detrimental impacts [10,11,43,44]. The alleviatory effects of Si against NH_4_^+^ toxicity are well documented in many plant species, such as cauliflower [45], cucumber [46], tomato [47], and sugar beet [18], but the specific study on Si treatments in NH_4_^+^-stressed salvia to date is still incipient. Therefore, the main purpose of the current study was to determine whether Si can attenuate the NH_4_^+^ toxicity degree in salvia.

### 4.1. Si Promoted Plant Growth and Alleviated the NH_4_^+^ Toxicity Degree

Si is able to promote several desirable plant physiological and morphological processes, consequently enhancing the growth and yield [48,49]. In our trials, salvia plants significantly improved in the shoot- and root-related parameters, as well as the whole dry matter mass, with Si supplementation (Figure 1, Table 2). These data were consistent with the findings of Barreto, who showed beneficial effects of Si in cauliflower and broccoli, regardless of the NH_4_^+^:NO_3_^−^ ratio [45]. Likewise, the effect of added Si to a 50:50 NH_4_^+^:NO_3_^−^ solution on the salvia plants improved these traits to varying extents (Figure 1, Table 2). Thus, for the non-NH_4_^+^-stressed plants, the strategy of adding Si to fertilizer can promote better plant growth, thereby contributing to a more sustainable production [17,18,22,50].

As stated above, 100% NH_4_^+^ nutrition given to the salvia plants showed not only a poor growth performance but also the development of typical NH_4_^+^ toxicity symptoms, as characterized by leaf necrosis, chlorosis, and stunted roots (Figure 1, Table 2, Figure 2A), which agrees well with our previous reports that found that greenhouse-grown salvia was also sensitive to high NH_4_^+^ levels [6,15].

Yet, as the most crucial part, Si is believed to reinforce the tolerance against abiotic stresses induced by NH_4_^+^ toxicity [17,50]. As expected, the NH_4_^+^-stressed plant growth was ensured and the manifested NH_4_^+^ toxicity degree was visibly reduced after the Si supplementation (Figure 2A). Concomitantly, the injured leaves and roots were also drastically ameliorated (Figure 1B–D). Consequently, the added Si significantly decreased the appearance of NH_4_^+^ toxicity symptoms as compared with the Si-deprived plants (Figure 2B). These data indicated that the NH_4_^+^ toxicity degree in salvia could be alleviated by the added Si.

### 4.2. Si Ameliorated Damaged Photosynthetic Capacity Caused by NH_4_^+^ Toxicity

Chlorophyll is an integral part of the photosynthetic reaction; it plays an important role in the absorption and transformation of light to chemical energy in support of the CO_2_ fixation [51]. Plants that possess higher chlorophyll contents are usually susceptible to exhibiting a greater light reaction rate in photosynthesis [6,52,53]. Moreover, the ratio between the variable chlorophyll fluorescence (Fv) and maximum fluorescence (Fm), namely, ‘Fv/Fm’, is a key indicator of the PSII (photosystem II) capacity, which is positively correlated to the quantum yield efficiency [54]. Nutrient-stimulated stresses can limit the photosynthetic ability mainly by damaging the PSII, conferring that Fv/Fm can be considered as a necessary criterion of early dynamic photoinhibition stresses [55,56]. Thus, the determinations of chlorophyll level and fluorescence Fv/Fm indicated the overall photosynthetic capacity.

In the present study, salvia seedlings displayed notably declined photosynthesis under NH_4_^+^ stresses, as characterized by the diminished chlorophyll content and Fv/Fm value (Figure 3). Numerous pioneering reports in cabbage [9,50], lettuce [9], and tomato [57] also revealed impaired photosynthetic capacity under a high NH_4_^+^ supply. Nevertheless, the Si-treated salvia plants had effectively enhanced chlorophyll contents and Fv/Fm values, irrespective of the NH_4_^+^:NO_3_^−^ ratio (Figure 3). The elevated leaf area after Si supplementation possibly improved the light interception, presenting a higher photosynthetic rate and CO_2_ assimilation [17,58]; augmented stomatal growth following Si supplementation, which can assist in the proper gas exchange and maintain the water status [17,18,45]; and the deposition of Si is able to enhance the protection of the photosynthetic pigments [17,59]. Succinctly, Si can help to improve the photosynthetic ability of salvia plants under NH_4_^+^ stresses.

### 4.3. Si Alleviated the Inhibition of Key Cation Uptakes under NH_4_^+^ Toxicity

To determine the uptake of Si and the quadratic effects on the regulations of other key ions, we further individually determined the K, Ca, Mg, and Si contents in the plant tissues from different treatments. It is evident that the external Si supply can significantly improve the internal Si level in plants (Figure 4A), exhibiting a similar Si absorption rate to many species [18,45,46,47], which indicated that salvia is a Si-accumulating species [60,61]. It is noteworthy that only minor fluctuations (no statistical differences) of Si content among different N treatments were observed, regardless of the Si-sufficient or -deficient plants (Figure 4A), which showed a negligible effect of the N form on the Si uptake in salvia.

Excessive NH_4_^+^ presented to the plants could instigate a nutritional disequilibrium due to the antagonistic impact of NH_4_^+^ against the uptake of other inorganic cations, particularly K^+^, Ca^2+^, and Mg^2+^. Therefore, a large amount of cation extrusion is a key marker of NH_4_^+^ toxicity [10,50,62]. Indeed, significant reductions in the contents of K and Ca in Si-deficient plants were observed when the external NH_4_^+^ supply increased from 0 to 100% (Figure 4B). Our data were in accordance with previous findings, displaying remarkably diminished K^+^, Ca^2+^, and Mg^2+^ in cauliflower, broccoli, and cabbage cultivated with 100% NH_4_^+^ [45,50]. Si supplementation of NH_4_^+^-stressed salvia plants distinctly reduced the extrusions of K and Ca (Figure 4B). Furthermore, greater K, Ca, and Mg contents were found in the plants cultured with 50:50 NH_4_^+^:NO_3_^−^ solutions with Si supplementation (Figure 4B), which may explain why the Si-sufficient plants had a better growth performance (Figure 1).

Si-treated salvia plants growing in the 100:0 NH_4_^+^:NO_3_^−^ regime had higher contents of K relative to their Si-deprived counterparts, probably due to the finding that the inclusion of Si could improve the activity of membrane-located H-ATPase, which is a primary transporter in the K acquisition and regulation processes [63,64]. The uptake and distribution of NH_4_^+^ were antagonistically modulated by K [15,65]. Accordingly, the alleviation of NH_4_^+^ toxicity may stem from the modulation of Si on K.

### 4.4. Improved Antioxidative Enzyme Activities by Si Contributed to the Mitigation of NH_4_^+^ Toxicity

Generally, the balance between the production and detoxification of ROS (mainly by O_2_^·−^ and H_2_O_2_) can be disturbed when plants are under abiotic stresses [66]. NH_4_^+^ toxicity-induced osmotic changes can boost the excessive accumulation of O_2_^·−^ and H_2_O_2_, accordingly resulting in higher lipid peroxidation and pigment degradation [11,17,62]. Plants have developed sophisticated strategies to tightly regulate the steady-state levels of ROS, such as antioxidant enzymes. As depicted in Figure 5, SOD was the most important and first line of defense that directly scavenged the O_2_^·−^ into H_2_O_2_, and subsequent rapid decomposition of H_2_O_2_ to water was aided by APX, CAT, and GPX [67]. Concurrently, regarding the ascorbate–glutathione (ASA-GSH) metabolizing cycle, the synergistic action regarding APX and GPX interacting with DHAR and GR, respectively, further contributes to the conversion of H_2_O_2_ to water [67,68]. Moreover, APX converts H_2_O_2_ into H_2_O by employing ASA (ascorbate) as an electron donor, while GPX decomposes the H_2_O_2_ to H_2_O using GSH (glutathione) [67,69].

Consistently, in the absence of Si, the antioxidative enzyme activities were amplified to varying extents when the external NH_4_^+^ supply increased from 50 to 100%, except for APX (Figure 5), illustrating that NH_4_^+^-stressed salvia adopted a species-specific strategy in the lowering of NH_4_^+^ toxicity [68]. Conspicuously, SOD, CAT, GPX, and DHAR activities in solely NH_4_^+^-treated plants were significantly enhanced as compared with the plants cultured with the 50:50 NH_4_^+^:NO_3_^−^ solution (Figure 5A,C,D,F).

Numerous studies showed that Si improved the antioxidative defense capacity to reduce the excessively produced ROS under abiotic stresses [17,50,70,71]. Indeed, the added Si distinctly promoted the antioxidative ability as characterized by the activated activities of antioxidative enzymes in the presented metabolism route (Figure 5). Outstandingly, SOD was the first sensing line of ROS, and thus, possessed the most reinforcements (Figure 5A), meaning that SOD is extremely important in the rapid scavenging of O_2_^·−^ in salvia [72]. Therefore, Si can enhance the antioxidative capacity, as evidenced by boosting the activities of antioxidative enzymes, especially in NH_4_^+^-stressed plants, accordingly indicating a tight regulation by Si against NH_4_^+^ toxicity-induced ROS metabolism. It can be assumed that the improved antioxidative enzyme activities by Si contributed to the mitigation of the NH_4_^+^ toxicity degree. Previous reports regarding cabbage [50], cucumber [73], bamboo [74], and wheat [75] also advocated for the beneficial impacts of Si on the antioxidant machinery.

### 4.5. Si Decreased the ROS Accumulation, Lipid Peroxidation, and Pigment Degradation

As depicted above, a disproportionate accumulation of ROS could oxidize the membrane lipids and photosynthetic pigments, causing an upsurge in MDA and a decrease in chlorophyll and carotenoid contents [17,50,76]. Consistent with this, for Si-deficient plants, solely NH_4_^+^-treated salvia rapidly intensified the levels of O_2_^·^^−^ and H_2_O_2_ while significantly decreasing the carotenoid and chlorophyll contents (Figure 3A and Figure 6), demonstrating that the integrity of the cellular membrane was damaged by NH_4_^+^ toxicity. The MDA content can directly reflect the cell membrane injuries [25,77]. Similar findings were previously acquired in *Hydrilla verticillata* [78] and rice [79].

Meanwhile, in line with many previous studies [32,41,50,80], we also showed that the supplementation of Si decreased the ROS content and MDA level as a consequence of increased antioxidative enzyme activities (Figure 6A–C). Moreover, degradation of chlorophyll and carotenoid contents were markedly truncated after the application of Si, except in solely NO_3_^−^-treated salvia plants (Figure 3A and Figure 6D). It is worth noting that the involvement of Si contributed to the decline of ROS accumulation, lipid peroxidation, and pigment degradation, which could be a modulation strategy that is involved in the mitigation of the NH_4_^+^ toxicity mechanism. In addition, the PCA data further exhibited the distinguished differences in the antioxidative defense system between the Si-deficient and Si-sufficient salvia plants, regardless of the supplied N form (Figure 7). These results suggested that Si could also enhance the antioxidative enzyme activities and concomitantly diminish the antioxidative injuries, especially in NH_4_^+^-stressed salvia.

## 5. Conclusions

To sum up, the damage of *Salvia splendens* raised in a controlled environment that introduced NH_4_^+^ toxicity manifested as seriously declined plant growth (biomass, shoot length, leaf length and width, leaf area, tap root length) and decreased photosynthetic ability (chlorophyll, quantum efficiency, and carotenoids). Similarly, NH_4_^+^-stressed salvia seedlings showed lower cation contents (K, Ca), together with increased synthesis of MDA and ROS. Conversely, the supplementation of Si significantly alleviated the NH_4_^+^ toxicity degree. This alleviation potential conferred by Si may be ascribed to the ameliorated photosynthesis, increased cation uptake (especially K), diminished lipid peroxidation (MDA), and enhanced performance of the antioxidative machinery to scavenge the over-production of ROS. It was accordingly concluded that the NH_4_^+^ toxicity could be mitigated by Si in *Salvia splendens*.

In addition, acidic stress caused by excessive NH_4_^+^ assimilation was elucidated as the primary cause of NH_4_^+^ toxicity. Therefore, further molecular study on the interaction between Si and H^+^ under high NH_4_^+^ conditions appears mandatory to interpret how Si assists in the alleviation of NH_4_^+^ toxicity.

## Figures and Tables

**Table 1:** Compositions of the nutrients (in me·L^−1^) for the treatment solutions used in this study.

Nutrient Source	Ammonium to Nitrate Ratio Combined with (+) or without (−) Si					
	0:100 Si (−)	0:100 Si (+)	50:50 Si (−)	50:50 Si (+)	100:0 Si (−)	100:0 Si (+)
NH_4_H_2_PO_4_	-	-	2.0	2.0	-	-
(NH_4_)_2_SO_4_	-	-	4.5	4.5	13.0	13.0
K_2_SO_4_	-	-	4.5	3.5	1.2	0.2
CaCl_2_·6H_2_O	-	-	-	-	4.9	4.9
Ca(NO_3_)_2_·4H_2_O	6.9	6.9	5.9	5.9	-	-
KNO_3_	4.8	3.8	-	-	-	-
Mg(NO_3_)_2_·6H_2_O	1.3	1.3	0.6	0.6	-	-
MgSO_4_·7H_2_O	1.0	1.0	1.4	1.4	1.7	1.7
KH_2_PO_4_	1.0	1.0	-	-	2.0	2.0
K_2_SiO_3_	-	1.0	-	1.0	-	1.0

**Table 2:** Responses of the dry weight, shoot length, and leaf length and width to the three NH_4_^+^:NO_3_^−^ ratios and Si application.

NH_4_^+^:NO_3_^−^ Ratio (A)	Si Supply (B)	Dry Weight (mg)	Shoot Length (cm)	Leaf Length (cm)	Leaf Width (cm)
0:100	−	10.1 ^z^	2.4	2.4	0.9
	+	11.0	2.5	3.0	1.0
50:50	−	10.3	2.3	2.9	0.9
	+	11.9	2.5	3.0	0.9
100:0	−	5.6	1.8	1.2	0.5
	+	7.9	2.2	1.9	0.7
*F*-test	A	** ^y^	***	*	**
	B	**	**	*	*
	A × B	*	***	***	***

^z^ Mean data are displayed within each column. ^y^ *, **, and *** indicate *p* < 0.05, <0.01, and <0.001, respectively, which were generated from a two-way ANOVA using the *F*-test.
